# An Alignment Free Framework for Taxonomic Inference From Codon and Codon‐Pair Usage

**DOI:** 10.1002/ece3.73169

**Published:** 2026-03-23

**Authors:** Sharon Yalov Handzel, Brian Rikshpun, Nigam Padhiar, Nathan Clement, Chava Kimchi‐Sarfaty

**Affiliations:** ^1^ Department of Machine Learning and AI Afeka Tel‐Aviv Academic College of Engineering Tel‐Aviv Israel; ^2^ Hemostasis Branch 1, Division of Hemostasis, Office of Plasma Protein Therapeutics CMC, Office Therapeutic Products, Center for Biologics Evaluation and Research, US Food and Drug Administration Silver Spring Maryland USA

**Keywords:** alignment‐free, classification, clustering, codon usage, codon‐pair usage, machine learning, taxonomy

## Abstract

Alignment‐free signals in coding sequences provide a scalable route to taxonomic inference, quality control of large phylogenies, and rapid screening of genomic data. We present a model‐agnostic framework that represents genomes or coding‐sequence collections using codon usage (64‐D) and codon‐pair usage (4096‐D) profiles, and we introduce Taxonomic Consistency (TC), a simple, rank‐aware external index, for evaluating supervised predictions or unsupervised clustering against the hierarchical taxonomy. Across multiple taxonomic ranks (e.g., Domain, Order, etc.), compact supervised models and standard clustering methods are assessed with both internal (Silhouette) and external (TC) validation. In large‐scale experiments, handling class imbalance and applying principled normalization had a greater impact on performance than sequence‐level preprocessing, and codon usage profiles yielded the highest TC and coherent unsupervised structure, while codon‐pair features provided complementary resolution within specific clades. We release open, versioned code and reproducible workflows to facilitate adoption in ecological and evolutionary pipelines. Taken together, alignment‐free codon and codon‐pair representations, paired with TC for transparent external validation, offer a practical complement to tree‐based methods—especially for rapid screening, sanity checks, and exploratory analyses at higher ranks.

## Graphical abstract

We present a model‐agnostic, alignment‐free framework that encodes genomes via codon (64‐D) and codon‐pair (4096‐D) usage profiles, and introduce Taxonomic Consistency (TC), a simple rank‐aware index for evaluating supervised and unsupervised methods against hierarchical taxonomy. In large‐scale experiments across multiple taxonomic ranks, we find that handling class imbalance and applying principled normalization influence performance more than sequence‐level preprocessing, with codon usage yielding the most coherent structure and codon‐pair features adding complementary resolution within specific clades.

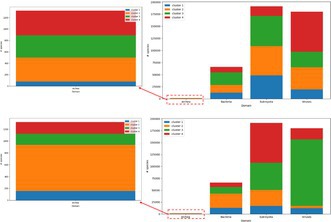

## 1 Introduction

Accurate taxonomic inference is fundamental to ecological and evolutionary research, from biodiversity surveys to phylogenomic studies. Traditional approaches rely on sequence alignment and tree reconstruction methods that, while powerful, face computational and methodological challenges when applied to large‐scale genomic datasets. Alignment‐based workflows require careful gene selection, multiple sequence alignment optimization, and sophisticated phylogenetic reconstruction—steps that become computationally prohibitive and sensitive to methodological choices as datasets grow to encompass 1000s of taxa.

The challenge of developing unified taxonomic systems has persisted since Darwin and Wallace first formulated evolutionary theory in the mid‐19th century (Freeman and Herron 2014). Modern taxonomy encompasses multiple systems: Linnaean classification based on morphological traits (Schoch 2011), evolutionary taxonomy that reconciles overall similarity with phylogenetic data, and phylogenetic taxonomy that groups organisms strictly by evolutionary descent (Hall 2013; Kapli et al. 2020). Despite advances in cladistics and DNA analysis (Allard et al. 2004; Yang and Rannala 2012), unresolved issues remain, including differing species concepts, representation of reticulate processes like horizontal gene transfer, and conflicting phylogenetic signals (Hey et al. 2003). While phylogenomics has improved evolutionary models by integrating genome‐scale data (Edwards et al. 2016; Philippe et al. 2005), computational demands continue to limit scalability.

Recent advances in genomic sequencing have generated unprecedented volumes of coding sequence data across diverse organisms, creating opportunities for scalable, alignment‐free approaches to taxonomic inference. Codon usage patterns, shaped by the interplay of mutation, selection, and genetic drift, contain phylogenetic signals that can be leveraged without the computational overhead of sequence alignment. Lin et al. (2002) demonstrated that ribosomal protein genes exhibit conserved codon composition across species, suggesting functional constraints maintain certain usage patterns across taxonomic boundaries. Building on this foundation, Miller, McKinnon, Whiting, Kauwe, and Ridge (2020) provided compelling evidence that codon pairs are phylogenetically informative, showing that codon‐pair usage patterns contain hierarchical signals correlating with established taxonomic classifications. Their subsequent work revealed that codon preferences are largely shaped by phylogenetic history rather than purely functional constraints (Miller, McKinnon, Whiting, and Ridge 2020).

More sophisticated machine learning approaches have expanded codon‐based phylogenetic inference capabilities. Hallee and Khomtchouk (2023) developed supervised learning methods for taxonomic classification using codon usage bias, achieving 91%–94% accuracy across taxonomic levels with curated orthologous gene datasets. Gupta et al. (2023) employed parsimony‐based methods using codon composition data, demonstrating these approaches can complement traditional sequence‐based phylogenetic methods. Recent applications have shown promise for identifying organelle origin and taxonomic identity across 1000s of organisms (Veisi 2023).

Despite these promising results, existing codon‐based methods face limitations for large‐scale applications. Most approaches focus on specific gene sets or require supervised training on curated datasets (Hallee and Khomtchouk 2023), limiting their scalability and generalizability. Furthermore, existing validation frameworks often rely on internal clustering metrics that may not capture alignment with established taxonomic hierarchies, making it difficult to assess performance in biologically meaningful terms (Chor and Tuller 2005; Fawcett 2006).

Here we introduce a general, alignment‐free framework for taxonomic inference that addresses these gaps through genome‐wide codon and codon‐pair usage analysis. Our approach makes four key contributions: (i) a unified representation using both codon usage (64‐dimensional) and codon‐pair usage (4096‐dimensional) vectors that captures short‐range compositional signals in coding sequences; (ii) a simple external validity criterion, Taxonomy Closeness (TC), specifically designed to evaluate clustering results against hierarchical taxonomic structure; (iii) a scalable evaluation protocol that demonstrates performance across multiple taxonomic ranks from Domain to Order; and (iv) an open, reproducible implementation with versioned code and standardized workflows suited to ecological and evolutionary pipelines.

We evaluate this framework using the FDA Codon and Codon‐Pair Usage Tables (CoCoPUTs) dataset (Alexaki et al. 2019), which provides comprehensive codon statistics across 100s of 1000s of organisms. This dataset has the potential to disentangle the evolutionary processes that shape the encoding of genetic information across diverse lineages, offering valuable insights for reconstructing branching patterns in molecular sequence‐based taxonomies (Pawlowski et al. 2005; Fu et al. 2020; Joiret et al. 2023).

Our machine learning approaches include Support Vector Machines (Su and Zhang 2006), Decision Trees (Pal 2005), Random Forests (Peng et al. 2002), and Logistic Regression (Ahmed et al. 2020) for supervised classification, and K‐Means clustering (Kohonen 1990) and Self‐Organizing Maps (Shahapure and Nicholas 2020) for unsupervised analysis. Performance evaluation combines established metrics (Silhouette index; Yeung et al. 2001) with our novel TC score to assess alignment with taxonomic hierarchies. Through both supervised classification and unsupervised clustering experiments, we demonstrate that alignment‐free codon‐based representations can achieve strong performance while remaining computationally efficient. Our results show that this approach provides a practical complement to tree‐based methods, particularly valuable for rapid screening, quality control of large phylogenies, and exploratory analyses at higher taxonomic ranks.

The framework is model‐agnostic and flexible, supporting multiple machine learning approaches while maintaining computational efficiency essential for large‐scale ecological and evolutionary applications. By providing standardized tools and validation metrics, this work aims to facilitate broader adoption of alignment‐free methods in the research community, offering a scalable alternative for taxonomic inference in an era of rapidly expanding genomic datasets.

## 2 Methodology

### 2.1 The Data

We used the FDA Codon and Codon Pair Usage Tables (CoCoPUTs) dataset (Alexaki et al. 2019), which provides per‐organism codon and codon‐pair counts along with NCBI Taxonomy annotations (Schoch 2011). Codon usage and codon‐pair usage have been widely used as compositional genomic signatures in comparative and alignment‐free analyses (Lin et al. 2002; Miller, McKinnon, Whiting, Kauwe, and Ridge 2020; Miller, McKinnon, Whiting, and Ridge 2020; Gupta et al. 2023). We worked from a fixed snapshot (date: 2021‐09‐01) to ensure reproducibility.

We retained entries with valid taxonomic labels at the ranks used in this study (Domain, Kingdom, Phylum, Class, and Order). Exact duplicates were removed. Because viral taxonomy follows distinct conventions and rank coverage differs from cellular life, the main analysis focuses on Archaea, Bacteria, and Eukarya. Viral organisms are summarized separately.

GC% was included as a simple, global compositional feature for two reasons. First, GC content captures broad mutational biases and genome‐wide base composition, which are known to covary with codon usage patterns and phylogenetic lineages (Lin et al. 2002; Miller, McKinnon, Whiting, Kauwe, and Ridge 2020). Second, using GC% alongside codon and codon‐pair profiles allows us to gauge whether taxonomic signal arises purely from detailed codon composition or is partly driven by more general GC bias.

GC% is defined here as a *relative* measure, that is, the proportion of G and C nucleotides among all nucleotides considered, rather than a raw count. As such, GC% is largely independent of sequence length: genomes of different lengths but with similar underlying nucleotide composition will exhibit similar GC%. Sequence length mainly affects the statistical stability of the estimate (shorter sequences yielding noisier GC% values), not its definition.

**FIGURE 1:**
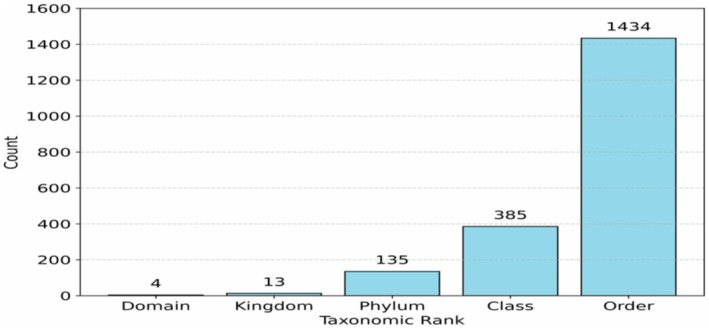
Number of categories for each of the first five ranks, based on NCBI taxonomy. This includes information about viral taxonomy as well.

After filtering, the dataset comprised 471,520 genomes in total: 1415 Archaea, 72,070 Bacteria, 204,895 Eukarya and 194,555 viruses. Figure 1 visualizes rank distributions for the cellular domains.

For each genome we formed the following features in 2 distinct datasets:

- Codon usage (64‐D)– counts of the 64 triplets within annotated coding sequences (CDS), converted to relative frequencies by L1 normalization.
- Codon‐pair usage (4096‐D) – L1 normalized counts of adjacent codon bigrams within CDS.

Using relative frequencies rather than raw counts yields length‐normalized compositional “signatures” for each genome or coding‐sequence collection. These signatures describe the preferences for particular codons and codon‐pairs, which are shaped by mutation, selection, and other evolutionary forces and are known to differ systematically among taxa. The resulting 64‐dimensional (codon) and 4096‐dimensional (codon‐pair) frequency vectors form the input features for our supervised classifiers and clustering algorithms, allowing the models to learn taxon‐specific patterns in codon usage. Using relative frequencies as features follows earlier work showing that such usage patterns embed phylogenetic and functional signal across diverse taxa (Lin et al. 2002; Miller, McKinnon, Whiting, Kauwe, and Ridge 2020; Miller, McKinnon, Whiting, and Ridge 2020).

To stabilize rare events, we applied an additive pseudo‐count *α* = 1 before normalization for both codon and codon‐pair counts. We also computed simple baselines: GC% and sequence length (ORF‐weighted).

Finally, we derived per‐genome Shannon entropies over the distributions of codons and codon‐pairs using

$$H=-\sum_{i}p_{i}\;\log p_{i}$$

where $p_{i}$ denotes the relative frequency of codon i(or codon‐pair i) for a given genome. Thus, H summarizes how “spread out” the codon (or codon‐pair) usage is: higher values indicate more uniform usage across codons, whereas lower values indicate a stronger preference for a subset of codons. We used these entropy values as summary statistics of dispersion but did not substitute them for the full codon or codon‐pair distributions in the main models.

Unless noted, models consumed normalized frequencies directly. For algorithms sensitive to feature scale (e.g., SVM, logistic regression), we applied standardization (zero mean, unit variance) per feature using training‐set statistics only.

We performed an 80/20 split at the species level so that strains of the same species do not appear across training and test sets. Splits were stratified by the target rank to mitigate class imbalance at that rank. Because closely related taxa can still induce information leakage across fine ranks, we mark family‐blocked evaluation as future work and report single‐split results here. When predicting multiple ranks, the split was defined once and reused for all ranks to ensure comparability.

### 2.2 Supervised Learning Processing

We implemented four supervised learning algorithms to model the relationship between codon/codon‐pair distributions and taxonomic classifications. These methods served as a benchmark to assess the strength of the correlation between codon and codon‐pair distribution data in various species and their respective positions in evolutionary taxonomy. We used established taxonomic classifications as our target variable, focusing on major taxonomic ranks such as domain, kingdom, phylum, class, and order.

To achieve this, we employed SVM, Decision Tree, Random Forest and Logistic Regression models.

Each of these models was chosen due to its ability to reveal other types of hidden correlations between the features and the corresponding label. The SVM model was selected for its ability to capture potential complex, non‐linear relationships in the data. Decision Trees were chosen due to their interpretability and capacity to capture hierarchical decision rules, which may align well with taxonomic hierarchies. Random Forest, an ensemble method, was included to potentially improve upon the performance of individual decision trees and to assess feature importance. Logistic Regression, a linear model, served as a baseline and allowed us to evaluate the presence of linear relationships between codon/codon‐pair usage and taxonomic classification.

For each model, we performed hyperparameter tuning using grid search with cross‐validation to optimize performance. Thereafter, a comparative analysis of the performance of each model was conducted. By employing this diverse set of machine learning methods, each with its own characteristics, we aimed to gain a comprehensive understanding of the complex relationships between genetic features and evolutionary taxonomy.

Supervised classification was performed across multiple taxonomic levels, including Domain, Kingdom, Phylum, Class, and Order. The strongest and most consistent predictive performance was observed at the Domain level, which informed the focus of our unsupervised clustering approach.

### 2.3 Unsupervised Learning Analysis

The underlying assumption is that evolution is a continuous process, and as a result, the distribution of codons and codon pairs in organisms with evolutionary kinship will exhibit similarities. This forms the basis for employing clustering algorithms to identify these similarity groups.

To facilitate a robust comparison and validate the presence of a consistent underlying taxonomic substructure through machine learning, we employed two distinct unsupervised clustering algorithms: K‐Means and Self‐Organizing Maps (SOM). These algorithms were chosen because they operate on different principles and are sensitive to hyperparameter and distance metric selection, providing complementary perspectives on the data structure.

K‐Means is a widely used algorithm that partitions observations into K clusters based on each data point's proximity to the cluster centroid, which represents the mean of all points in that cluster. The algorithm operates iteratively, assigning data points to the nearest cluster and recalculating the cluster centroids until convergence or until a maximum number of iterations is reached. The number of clusters, K, is a crucial hyperparameter for this algorithm, and its performance is sensitive to both the choice of K and the initial placement of the centroids.

SOMs, on the other hand, are unsupervised neural networks used for dimensionality reduction and clustering. SOMs maintain a topological structure where similar data points are mapped to adjacent neurons in the output layer. The neuron in the output layer compete to represent the input data, and the winning neuron and its neighbors are updated to become more like the input. The hyperparameters of SOMs include the network architecture (i.e., number of neurons, neighborhood function and learning rate), which can significantly impact their performance.

### 2.4 Evaluation Metrics

To comprehensively assess the performance of both supervised and unsupervised learning models applied to codon and codon‐pair datasets, we utilized a range of evaluation metrics. For the supervised learning models, we primarily relied on metrics derived from the confusion matrix, a tabular summary of a model's classification performance that displays the counts of true positives, true negatives, false positives, and false negatives (Ahmed et al. 2020). The main focus was on accuracy, which provides an overall measure of correct classifications across all taxonomic levels.

For unsupervised learning methods we used the Silhouette score (Shahapure and Nicholas 2020) which is a common metric to estimate clustering in terms of cohesion and separation. It measures how similar an object is to its own cluster compared to other clusters, in terms of Euclidean distance. The score ranges between −1 and 1, where higher values indicate a good match of each point to its own cluster and a poor match to other clusters.

To assess the similarity between the clustering results and the reference NCBI taxonomy, we developed a novel metric called TC, an external validation index tailored to hierarchical taxonomies. Unlike standard external clustering indices such as adjusted Rand index (ARI), normalized mutual information (NMI), or purity, which assume a flat label space and treat all misassignments as equally severe, TC explicitly incorporates the structure of the taxonomic hierarchy. It compares the homogeneity of each node in the tree by the entropy of its ancestor nodes, while relating the corresponding node in the reference tree as having full homogeneity. The entropy of all leaves is zero. Then, the score of a node j which is one upper level in the tree, and that all its ancestors are leaves is calculated as NE(j) in Equation (1). The TC score is calculated as follows:

- For each node in the clustering tree, calculate the Node Entropy (NE):

(1) $$\mathrm{NE}=-\Sigma \left(p\left(i\right)\times \log\left(p\left(i\right)\right)\right)$$

where *p*(*i*) is the probability of species that belong to node *i* in the reference taxonomy being an ancestor of the current node.

b Propagate the NE scores upwards to the root, using a weighted sum:

(2) $$\mathrm{TC}\left(i\right)=\Sigma \left(w\left(j\right)\times \mathrm{NE}\left(j\right)\right)$$

where *j* represents the taxonomic rank, starting from the lowest rank up to rank *i*, and *w*(*j*) is the ratio of species classified under rank *j* compared to the total species under this rank.

**FIGURE 2:**
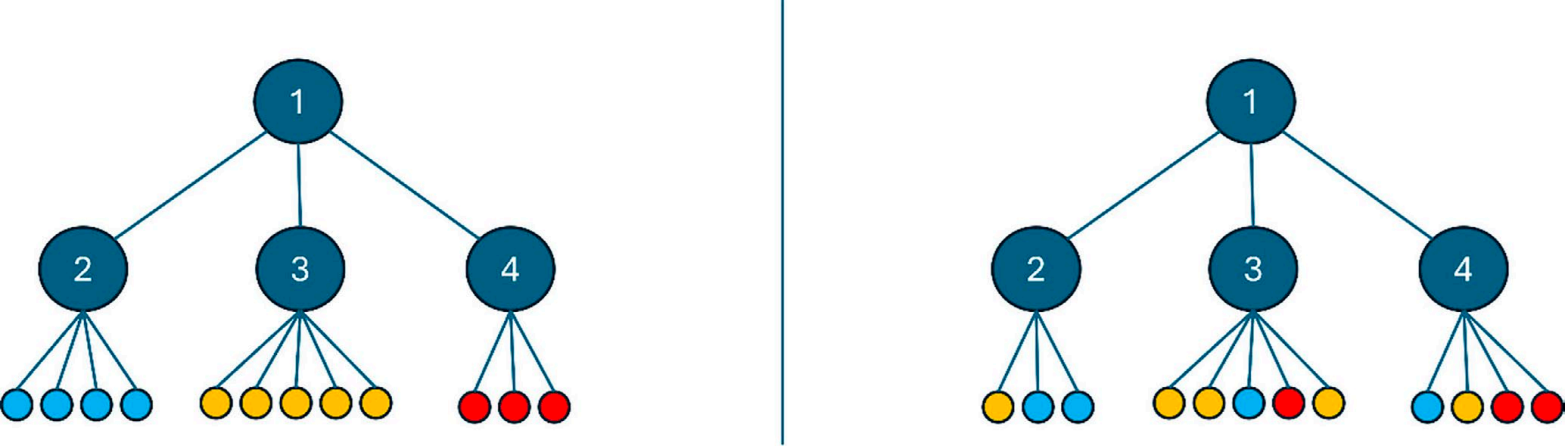
Depiction of two different taxonomy trees. On the left, we present an example of a reference taxonomic tree, and on the right is a taxonomic tree as constructed by our codon/codon‐pair analysis. The colors signify the different parent nodes in the reference hierarchy. This is used to explain the TC score calculation within the text.

The TC score is a non‐negative value, with values closer to zero indicating higher similarity to the reference taxonomy. Figure 2 shows an example of two taxonomies: a reference hierarchy on the left and a new taxonomy resulting from clustering, on the right. In the new taxonomy, categories are distributed across branches rather than organized into a distinct subtree. Entropy for each branch is calculated based on color distribution. Different colors denote the distinct parent nodes within the reference hierarchy. For example, in branch two, with two blue leaves (*p* = 2/3) and one yellow leaf (*p* = 1/3), the NE value is 0.918. For node 3 with 3 yellow, one blue, and one red ancestor, the NE value is 1.371. Node 4, with 2 red, 1 blue, and one yellow ancestor, the NE value is 1.5, according to the following calculation:

$$\mathrm{NE}\left(2\right)=-0.33\times \log0.33-0.67\times \log0.67=0.918$$

$$\mathrm{NE}\left(3\right)=-0.6\times \log0.6-2\times 0.2\times \log0.2=1.371$$

$$\mathrm{NE}\left(4\right)=-2\times 0.25\times \log0.25-0.5\times \log0.5=1.5$$

Then, calculating TC score is a weighted sum of these three entropies, which is equal to:

$$\mathrm{TC}=\left(3/12\right)\times \mathrm{NE}\left(2\right)+\left(5/12\right)\times \mathrm{NE}\left(3\right)+\left(4/12\right)\times \mathrm{NE}\left(4\right)=1.301$$

In addition to TC, we report the Silhouette coefficient as a standard *internal* clustering index. The Silhouette score evaluates the cohesion and separation of clusters in feature space without using taxonomic labels. Together, TC and Silhouette provide complementary views: Silhouette assesses the geometric quality of clusters induced by the alignment‐free representations, whereas TC assesses how well those clusters respect the hierarchical taxonomy at each rank.

The performance of the supervised analysis was measured by accuracy, which is a common metric that quantifies the proportion of correct predictions made by classification models. A prediction is considered correct when the model accurately classifies the location of species within the hierarchy subtree.

## 3 Results

### 3.1 Supervised Learning Results

**FIGURE 3:**
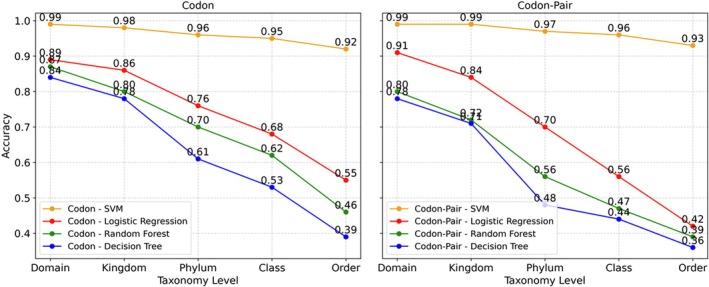
Supervised model accuracy for codon (left) and the codon‐pairs (right) datasets. Accuracy is reported here for every model at the first five taxonomic ranks.

We evaluated the performance of four supervised learning algorithms (SVM, Decision Trees, Random Forests, and Logistic Regression) on both codon and codon‐pair datasets across different taxonomic ranks. Figure 3 illustrates that the SVM model outperformed others, achieving the highest accuracy, which is the rate of correct predictions compared to the NCBI taxonomy. Notably, the results obtained using codon‐pair data surpassed those from codon data across all four models. Significantly, even on the *Order* rank, which encompasses 1434 different classes, the SVM model attained an impressive accuracy of approximately 0.92. This remarkable performance suggests that the supervised model effectively learned the relationship between the distributions of codons and codon‐pairs, and the corresponding position in the evolution taxonomy.

**FIGURE 4:**
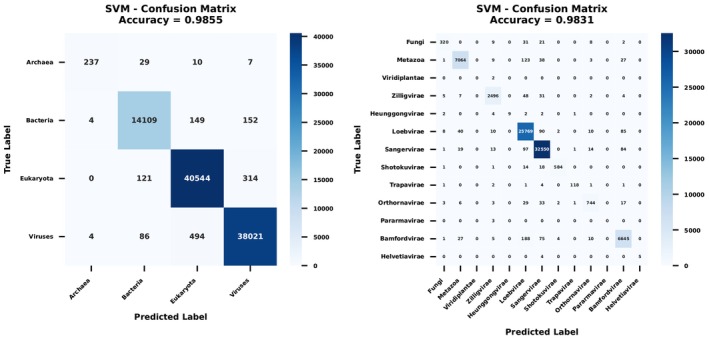
Confusion matrix for domain (left) and kingdom (right) ranks applied to the codon dataset.

Figure 4 illustrates the confusion matrix in both Domain and Kingdom as achieved by the SVM analysis applied to codon dataset.

The supervised learning results demonstrated that the Support Vector Machine (SVM) model achieved superior performance compared to other algorithms across all taxonomic ranks for both codon and codon‐pair data, while codon data generally produced higher accuracy scores than codon‐paired data across most models and taxonomic ranks; furthermore, a consistent decline in performance was observed for all models as the taxonomic rank progressed from Domain to Order, indicating the greater difficulty of classification at increasingly specific taxonomic levels.

### 3.2 Unsupervised Learning Results

**FIGURE 5:**
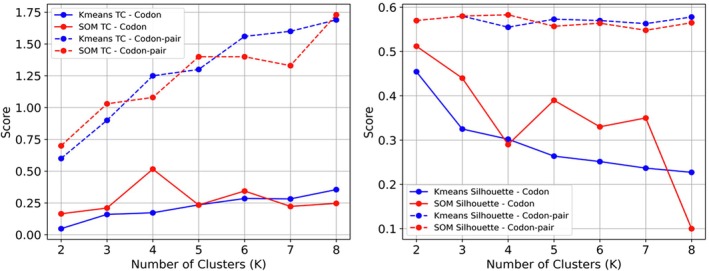
This figure presents the TC (left) and Silhouette score (right) for K‐Means and SOM clustering on both codon and codon‐pair data, across different numbers of clusters (K).

Two unsupervised clustering algorithms, K‐Means and SOM, were applied to both codon and codon‐pair datasets. The resulting clusters were evaluated using the Silhouette score and our novel TC score. These algorithms were executed with a varying number of clusters in the range 2–8, to explore the inherent structure within the data. The quality and validity of the resulting clusters were assessed using two distinct evaluation metrics: the Silhouette and the newly proposed TC score. The outcomes of these clustering experiments are presented in Figure 5. The Silhouette score measures the compactness and separation of clusters, while the TC score evaluates the correspondence between the identified clusters and the known taxonomic classifications. The Silhouette ranges between −1 and 1, where closer to 1 is considered better clustering. The TC can be any positive number, where the clustering is better closer to zero.

As shown in Figure 5, Silhouette scores summarize the geometric cohesion and separation of clusters in codon and codon‐pair feature space, while TC indicates how these clusters align with the known taxonomy. Consistently high TC together with reasonable Silhouette values indicate that the alignment‐free features form both geometrically meaningful and taxonomically coherent structures.

**FIGURE 6:**
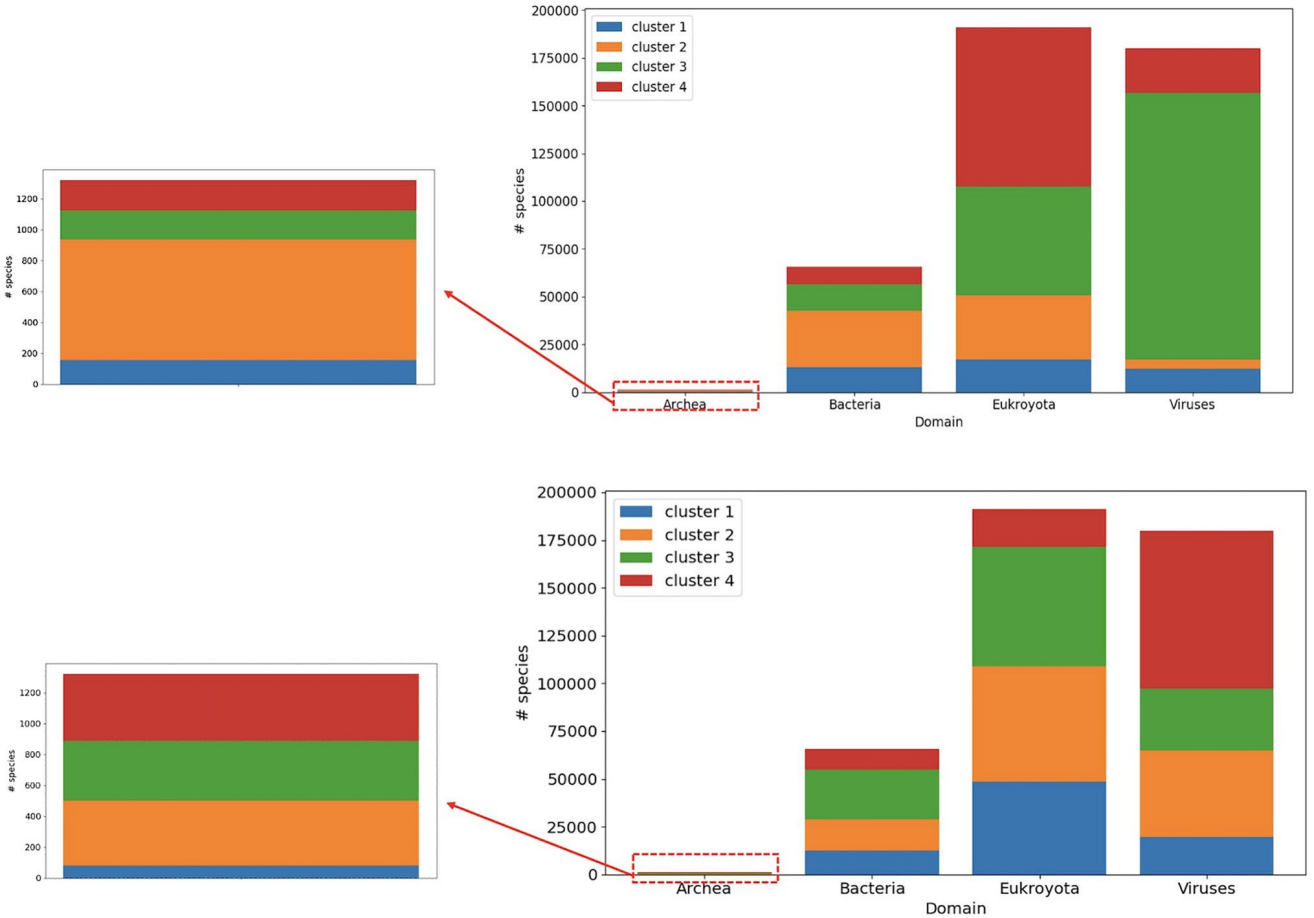
Clustering results of codon (lower) and codon‐pair (upper) data. Each bar represents the number of species assigned to a domain based on Linnean taxonomy—the shading represents the proportion of these species which are assigned to each cluster generated from codon/codon‐pair data. Codon clustering shows clearer domain separation. Archaea results are shown separately due to limited species.

Figure 6 presents the clustering outcomes based on codon frequencies (lower) and codon‐pair frequencies (upper). The codon‐based clustering exhibits clearer biological structure, with distinct clusters predominantly corresponding to known Domains: Cluster 2 largely captures Archaea and Bacteria, Cluster 3 represents Viruses, and Cluster 4 aligns with Eukaryota. Notably, this separation emerges despite the model being unsupervised and receiving no taxonomic input. In contrast, the clustering based on codon‐pair data appears less distinct, which may indicate that the higher‐dimensional codon‐pair features introduce complexity that the algorithm struggles to interpret effectively. These findings suggest that codon usage alone encodes biologically meaningful patterns that align with high‐level taxonomic classifications.

Our clustering quality analysis revealed several important patterns. Codon‐pair data consistently yielded higher Silhouette scores compared to codon data for both K‐Means and SOM clustering, indicating better‐defined clusters. Interestingly, codon data consistently produced lower TC scores, suggesting higher similarity to the NCBI taxonomy compared to codon‐pair data across both clustering methods. K‐Means clustering generally produced slightly higher Silhouette scores and lower TC scores than SOM, though these differences were minimal. We also observed that Silhouette scores decreased as the number of clusters increased, indicating that smaller numbers of clusters resulted in more distinct and well‐separated groupings.

## 4 Discussion and Conclusions

Our alignment‐free framework demonstrates strong performance for taxonomic inference across multiple hierarchical levels, with several key findings relevant to ecological and evolutionary applications. Across taxonomic ranks from Domain to Order, supervised classification performed well, and the superior performance of linear models over tree‐based approaches suggests that linear combinations of codon and codon‐pair features are more predictive than complex non‐linear relationships, supporting the framework's computational efficiency.

The consistently superior performance of codon‐pair features (4096‐dimensional) over individual codon usage (64‐dimensional) across both supervised and unsupervised tasks highlights the importance of sequential context in genomic signatures. This finding has practical implications for method selection: while codon usage provides valuable information with lower computational overhead, codon‐pair analysis captures additional evolutionary constraints related to translational efficiency, mRNA structure, and regulatory mechanisms that improve taxonomic resolution.

The TC metric successfully captures alignment between unsupervised clustering results and established hierarchical taxonomy, indicating substantial concordances with NCBI classifications at broader ranks. However, TC scores decrease as cluster numbers increase, confirming the hierarchical nature of evolutionary relationships and suggesting optimal performance at higher taxonomic levels. The divergence between high Silhouette scores (indicating well‐defined clusters) and variable TC scores reveals that codon‐based clustering may capture biological signal not fully represented in current taxonomic frameworks.

Our methodology faces several acknowledged limitations that affect broader applicability. Variability in genome assembly completeness within large‐scale databases introduces heterogeneity that may influence codon statistics and clustering behavior. The framework currently does not address missing or partial genomic data common in transcriptomic studies, though it performs robustly on curated, analysis‐ready genomes. Additionally, our focus on nuclear genomic data excludes organellar genomes with distinct evolutionary histories and codon usage patterns.

The framework's computational efficiency represents a significant advantage for large‐scale applications. Unlike alignment‐based approaches that require intensive sequence alignment and tree reconstruction, our method scales linearly with dataset size while maintaining consistent performance. This scalability is particularly valuable for quality control applications in phylogenomic pipelines, where rapid screening of 1000s of taxa can identify potential misclassifications or contamination before computationally expensive analyses.

The recursive application potential of our hierarchical clustering approach addresses taxonomic resolution beyond the Domain‐to‐Order scope demonstrated here. The strict hierarchical structure of biological taxonomy allows iterative refinement within taxonomic groups, enabling species‐level classification through successive clustering steps. This recursive strategy could be particularly valuable for ecological surveys requiring fine‐scale taxonomic resolution.

This work contributes three key methodological advances to the ecological and evolutionary toolkit. First, the unified codon/codon‐pair representation provides a standardized approach for genomic signature analysis that is independent of gene selection and alignment quality. Second, the TC validation metric offers an external criterion specifically designed for evaluating clustering results against established taxonomic hierarchies, addressing a significant gap in validation frameworks. Third, the open, reproducible implementation with versioned workflows facilitates adoption across diverse research applications.

Future methodological development could address current limitations through several approaches. Integration of additional genomic features such as gene synteny, regulatory elements, or conserved neighborhoods could enhance taxonomic resolution. Development of robustness assessments under simulated data loss would evaluate performance with incomplete genomic data. More sophisticated hierarchical classification metrics could refine the quantification of taxonomic similarity beyond the current TC implementation.

We present a scalable, alignment‐free framework for taxonomic inference that effectively leverages codon and codon‐pair usage patterns as phylogenetic signals. The framework demonstrates strong performance across multiple taxonomic levels while maintaining computational efficiency essential for large‐scale ecological and evolutionary applications.

This approach provides a practical complement to traditional tree‐based phylogenetic methods, particularly valuable for rapid screening, quality control, and exploratory analysis of large genomic datasets. This role of alignment‐free compositional methods as scalable complements to alignment‐based phylogenetics has also been highlighted in prior work on large genomic datasets (Hall 2013; Kapli et al. 2020; Chor and Tuller 2005). The open implementation and standardized workflows support broader adoption in research pipelines where computational efficiency and scalability are paramount.

As genomic datasets continue to expand, alignment‐free approaches will become increasingly valuable for preliminary analysis, hypothesis generation, and large‐scale taxonomic screening. The demonstrated effectiveness of codon‐based features suggests broader potential for compositional approaches to phylogenetic inference, opening avenues for further methodological development in computational phylogenetics and evolutionary genomics.

## Author Contributions

**Sharon Yalov Handzel:** data curation (equal), methodology (lead), validation (equal), writing – original draft (lead), writing – review and editing (equal). **Brian Rikshpun:** data curation (equal), methodology (equal), software (lead), validation (equal), visualization (lead), writing – original draft (supporting), writing – review and editing (equal). **Nigam Padhiar:** methodology (supporting), writing – review and editing (equal). **Nathan Clement:** methodology (supporting), writing – review and editing (equal). **Chava Kimchi‐Sarfaty:** conceptualization (equal), writing – review and editing (equal).

## Conflicts of Interest

The authors declare no conflicts of interest.

## Data Availability

All data used in this study are publicly available from doi: http://dx.doi/10.1016/j.jmb.2019.04.021 (Alexaki et al. 2019). The code used for analysis is available at https://github.com/BrianRikshpun/ET_paper_code.
